# Are Adolescent Athletes Sleeping Enough? An Observational Study of Sleep Parameters during Schooldays and Holidays

**DOI:** 10.3390/children11091044

**Published:** 2024-08-27

**Authors:** Mehdi J. Souabni, Maher Souabni, Omar Hammouda, Tarak Driss

**Affiliations:** 1 Interdisciplinary Laboratory in Neurosciences, Physiology and Psychology: Physical Activity, Health and Learning (LINP2), UFR STAPS (Faculty of Sport Sciences), Paris Nanterre University, 92000 Nanterre, France; s.mehdi@parisnanterre.fr (M.J.S.); maher.souabni.phd@gmail.com (M.S.); tarak.driss@parisnanterre.fr (T.D.); 2 Research Laboratory, Molecular Bases of Human Pathology, LR19ES13, Faculty of Medicine, University of Sfax, Sfax 3029, Tunisia

**Keywords:** adolescents, sleep patterns, sleep deprivation, health, basketball, athletes, sport, wearable sensors

## Abstract

**Background:** Inconsistent sleep schedules, frequent awakening after sleep onset (WASO), and decreased sleep efficiency (SE) are common issues among adolescent team sports athletes. Moreover, research indicates that sleep problems are enhanced across schooldays. The aim of the present study was to assess sleep patterns of adolescent athletes and compare sleep parameters between schooldays and holidays. **Methods:** The chronotype and sleep quality of twelve adolescent basketball players (mean age: 15.58 ± 0.67 years) were assessed. Objective sleep parameters were then analyzed using actigraphy over a 12-day period, which included six days during the school period and six days during holidays. **Results:** Data showed that total sleep time (TST), SE, and WASO (382.48 min, 81.81%, and 66.70 min, respectively) did not meet international recommendations for sleep quantity and quality. During school weekdays, time in bed (TIB), TST, and SE significantly decreased compared to weekends (*p* < 0.001, d = −1.49; *p* < 0.001, d = −1.64; and *p* = 0.01, d = −0.89, respectively). On weekdays, TIB, TST, and WASO were significantly lower on schooldays compared to holidays (*p* < 0.001, d = −1.83; *p* < 0.01, d = −1.01; and *p* = 0.02, d = −0.77, respectively). While no significant difference was observed in social jetlag, the mid-point of sleep was significantly later on holiday weekdays compared to school weekdays (*p* < 0.05, d = 0.65). **Conclusions:** Adolescent athletes experience insufficient sleep, especially on school weekdays, which is partially improved during weekends and holidays. Although sleep duration was longer during holidays, our results suggest that adolescent athletes’ sleep was more fragmented. Consequently, it remains crucial to implement strategies to enhance their sleep health (e.g., napping).

## 1. Introduction

Sleep is an essential component of normal growth and development during childhood and adolescence. Over the last few decades, researchers have highlighted the importance of acquiring adequate sleep and maintaining a sleep schedule that is consistent with physiological circadian rhythmicity [1,2]. Adequate sleep is necessary for optimal mental and physical alertness, daytime functioning [3], memory consolidation, and learning capacity in youth [4], qualities that are of particular importance in the school setting. Of note, sleep health in adolescents is influenced by several factors, including physical activity. Indeed, it has been reported that physical activity is linked to a lower risk of insomnia symptoms, shorter sleep latency, and a reduced risk of bruxism [5,6], a condition affecting 9% of youth according to a recent meta-analysis [7]. Moreover, research has demonstrated that sleep is paramount for athletes [8], allowing better physical [9], cognitive [10], and physiological [11] responses, especially in pediatric athletes whose minds and bodies are in the process of development while trying to succeed with academic and athletic performance in school [12]. Importantly, the National Sleep Foundation recommends 8–10 h of sleep per night for adolescents (14–17 years), 7–9 h in adults (18–64 years), and 7–8 h in older adults (≥65 years) [13]. Although longer sleep durations are recommended for youth compared to older adults, adolescents appear more vulnerable to short sleep durations [14]. Adolescence is a mental, physical, and social developmental period [15], wherein healthy sleep patterns are often neglected, leading to a higher prevalence of insufficient sleep among teens [16]. In this way, the sleep need for adolescents was estimated using dose–response modeling under experimental settings, and the results showed that approximately 9 h is needed for optimal functioning [17]. Yet, evidence suggests that adolescents, across countries and cultures, do not get these recommended nine hours of sleep per night [18,19]. In fact, a meta-analysis of 41 surveys studying the worldwide sleep patterns and problems during adolescence showed that this recommended duration is longer than what adolescents habitually obtain in Australia, Europe, Asia, and the United States [20], indicating a substantial sleep debt in this age group on an international scale.

In this context, a growing body of evidence suggests that insufficient sleep quality and duration in adolescents are strongly linked with negative outcomes in several areas of health and functioning, including somatic and psychosocial health, school performance, and risk-taking behavior [21,22,23]. Indeed, a higher sleep disturbance has been associated with increased markers of cardiovascular risk [24]. In addition, research has reported associations between short sleep duration and cardiometabolic markers [25], as well as impaired insulin resistance [26]. In this way, cross-sectional studies in adolescents have provided support for an association between short sleep and overweight and/or obesity [27,28]. Interestingly, an inverse relationship between sleep duration and both BMI and being overweight/obese has been reported. In fact, each one hour decrease in sleep time increased the likelihood for overweight or obesity by 6.5% [27]. When athletes are compared to non-athletes, they tend to experience less efficient and shorter sleep [29]. In fact, inconsistent sleep schedules, insufficient sleep duration, and poor sleep quality, including prolonged sleep onset latencies (SOLs), frequent awakening after sleep onset (WASO), and decreased sleep efficiency (SE), are prevalent problems among adolescent team-sport athletes [30,31,32,33]. Several sport-related factors affecting sleep have been identified, including training, competition, travel [34,35], and nutrition and energy status [36,37]. This insufficient sleep duration can impact not only metabolism and endocrine function, but also athletic and cognitive outcomes, increasing the perceived effort during exercise and even the risk of injury [37,38,39,40,41]. In the same vein, it has been widely documented that sleep deprivation affects endurance, strength, and speed performances; increases muscle damage; and impairs recovery from high-intensity exercise [9,10].

Moreover, findings indicate that adolescents experience sustained sleep debt across schooldays. The Youth Risk Behavior Survey found that 72.7% of high school students reported an average of <8 h of sleep on school nights [42]. Similarly, the National Sleep Foundation poll reported that 62% of students (11–17 years) get <8 h of sleep on weeknights. In this way, weekday-to-weekend sleep discrepancy is a common phenomenon in school-age youths [14,20,43]. Furthermore, weekends and holidays, where longer sleep duration and later wake times are permitted [44], appear to offer opportunities to reduce sleep debt. It has been reported that during these times, cumulative sleep debt decreases, and the self-reported sleep duration meets adolescents’ perceived sleep need [43]. This misalignment of adolescents’ weekday–weekend sleep pattern, coined social jetlag [45], may be explained by the mismatch between biological changes due to pubertal development and environmental demands [46,47]. Importantly, a recent systematic review of 72 observational studies reported that longer weekend catch-up sleep may be associated with depressive symptoms and poorer academic performance, and may increase the risk of overweight/obesity especially for youths who are often chronically sleep-deprived during weekdays [46].

Although some studies have investigated sleep habits among adolescent athletes, most have used subjective methods such as questionnaires to evaluate sleep. To our knowledge, no studies have evaluated weekday-to-weekend and schooldays-to-holidays sleep discrepancies in these athletes. Thus, the aim of the present study was to assess sleep patterns in adolescent athletes (i.e., basketball players) and compare sleep parameters between schooldays and holidays. We first hypothesized that sleep durations could be longer during holidays and weekends compared to schooldays. Our second hypothesis is that, on holidays, these athletes might experience better sleep quality with less awakening during the night compared to schooldays.

## 2. Materials and Methods

### 2.1. Participants

Twelve adolescent basketball players were recruited from a local basketball club and participated in the current study. They trained regularly 4 to 5 times/week (≈2 h/time) and played 1 match/week. Sample characteristics are shown in Table 1. The sample size was a priori calculated using G*power software (version 3.1.9.4) [48] and following the suggested procedure by Beck [49]. Based on an earlier study with a similar paradigm [9], the effect size was estimated to be 0.54. To reach the desired power (0.95), data from at least nine participants were deemed sufficient to minimize the risk of incurring a type 2 statistical error. Participants were totally informed about the experiment, the tools, and the questionnaires, and a written informed consent was obtained from all of them and their parents (or legal representatives). To reduce variance related to sex and age, the sample consisted of male basketball adolescents from secondary high schools aged between 15 and 17 years old (mean SD = 15.58 ± 0.67 years). They did not have a professionally diagnosed sleep disorder (per parent and self-report), did not use medication with known effects on sleep, and had no extreme chronotype, and they were asked to stay away from caffeinated beverages. This research was approved by the local Institutional Review Board (CPP SUD No 0339/2021) and was carried out according to the Declaration of Helsinki.

### 2.2. Procedure

At baseline, the participants were assessed for subjective sleep quality and chronotype and took part in habituation night sleeping with the portable objective sleep devices (GT3X, wrist activity monitor actigraph). Subjective sleep quality was assessed using the Pittsburgh Sleep Quality Index (PSQI) developed by Buysse et al. [50]. The PSQI comprises 18 self-report items that generate seven component scores, including subjective sleep quality, sleep latency, sleep duration, sleep efficiency, sleep disturbances, sleep medication usage, and daytime dysfunction. Each item is scored on a scale from 0 to 3, with higher scores indicating poorer sleep quality. The global PSQI score, ranging from 0 to 21, provides an overall assessment of subjective sleep quality. A global score greater than 5 is considered a sensitive and specific indicator of poor sleep quality [50,51]. The PSQI has demonstrated adequate reliability and validity in previous studies [51,52,53] and has been successfully applied to adolescent athletes [9]. Participants’ chronotype, indicating their preference for morning or evening activities, was evaluated using the Morningness–Eveningness Questionnaire (MEQ) developed by Horne and Ostberg [54]. This questionnaire, validated for use in adolescents [55], consists of 19 questions assessing preferences for waking and sleeping times as well as optimal times for various activities. Each response is assigned to a specific value, and an overall score is calculated. Based on this score, participants were classified into one of five chronotype categories: definitely morning type (score from 70 to 86), moderately morning type (score from 59 to 69), no specific type (score from 42 to 58), moderately evening type (score from 31 to 41), and definitely evening type (score from 16 to 30). Higher scores on the MEQ indicate a stronger preference for morning activities.

Then, objective sleep quality and quantity were measured using the actigraphy method. Participants were instructed to wear the GT3X actigraph on their non-dominant wrist, following the Standards of the American Academy of Sleep Medicine [56], continuously for two weeks while maintaining their usual sleep–wake patterns. The GT3X device is renowned for its ability to continuously monitor the rest–activity cycle across diverse populations [57]. Compared to polysomnography (PSG), which is the gold standard for sleep assessment, actigraphy is considered a valid and reliable tool [58], particularly for adolescents [59]. Furthermore, actigraphy has been established as the preferred method for objectively measuring sleep in athletic populations due to its minimal disruption to sleep and training routines [35]. Additionally, the young athletes completed a sleep diary to document their sleep–wake cycles, including the start and end times of sleep episodes as well as instances when the wrist actigraph was removed. Studies suggest that five or more nights of usable recordings are required to obtain reliable actigraph measures of sleep for adolescents [60,61]. Data of 12 days, of which 6 days were holidays and 6 days were schooldays, were analyzed using Actilife 6 software (version 6.13.7, Actigraph, Pensacola, FL, USA). Moreover, Monday, Tuesday, Wednesday, and Thursday were considered schooldays (as participants had to wake up on time to go to school), and Friday and Saturday were considered weekends, in order to evaluate weekend-to-weekdays discrepancies. The Actilife 6 software’s algorithm scored, with a validated high-sensitivity threshold, each one-minute epoch as either sleep or wake based on movement levels during that minute, relative to the two minutes before and after [62]. This software generated graphical records in the form of actograms for each athlete. Key variables analyzed by the device included bedtime (BT), rise time (RT), time in bed (TIB), total sleep time (TST), sleep onset latency (SOL), sleep efficiency (SE), and wake after sleep onset (WASO). SOL, representing the transition period between wakefulness and sleep onset, is deemed optimal when ≤15 min across all age groups, indicative of good sleep quality [63]. SE, calculated as the percentage of TST relative to the time spent in bed, is considered satisfactory when ≥85%, reflecting good sleep quality [63]. WASO measures sleep fragmentation, with values ≤20 min recommended for optimal sleep quality [63]. Separate analyses were performed for weekdays and the weekend on schooldays and holidays. Social jetlag was defined as the difference between the mid-point of sleep times (midsleep) on weekdays and on the weekend [45], with the Mid-point of sleep being the time of a halfway point between sleep onset and offset [64].

### 2.3. Statistical Analysis

The data were initially collected using an Excel^®^ data collection table (Microsoft Office, v. 2016), encompassing all study variables. Statistical analysis was conducted using SPSS Statistics software V25 (2017, Armonk, NY, USA: IBM Corp). Inferential statistical analysis was performed at a confidence level of 95%. Normality was assessed using the Shapiro–Wilk test to ensure the suitability of parametric tests. When normality assumptions were met, hypothesis testing was carried out using the paired Student *t*-test, accompanied by Cohen’s d for effect size estimation. In cases where the normality assumption was violated, the Wilcoxon test was used. To compare the means of sleep variables across different periods (i.e., weekdays/weekends and schooldays/holidays), two-way repeated-measures ANOVA [2-week (holidays and schooldays) × 2-day (weekdays and weekends)] was employed for normally distributed variables. For non-normally distributed variables, Friedman’s nonparametric analysis of variance was applied. Bonferroni’s post hoc test was conducted when necessary. Reported statistics include the mean, standard deviation (SD), standard error, degree of freedom, *F*-test values, significance levels (*p*), and partial eta-squared (ηp^2^) and Cohen’s difference (d) for effect size. ηp^2^ effect sizes were classified as small, medium, and large for values above 0.01, 0.06, and 0.14, respectively [65]. For Cohen’s d, values above 0.2, 0.5, and 0.8 represent small, moderate, and large effects, respectively [66].

## 3. Results

### 3.1. Subjective Sleep Quality and Chronotype

The subjective sleep quality score assessed using PSQI was 4.75 ± 1.42 with 50% of the athletes possessing a PSQI score equal or superior to 5. Furthermore, the overall average MEQ score among the sampled athletes was 42.58 ± 4.89. Notably, 41.67% of the participants were identified as moderately evening type. In the same vein, the bivariate analysis revealed a significant association between greater eveningness preference and poorer sleep quality (r = −0.68, *p* = 0.01).

### 3.2. Sleep Parameters on Holidays vs. Schooldays

The average TST, SE, and WASO during the experimental period were 382.48 ± 46.97 min, 81.81 ± 7.23%, and 66.70 ± 28.26 min, respectively. The paired *t*-test showed no significant difference in bedtimes on holidays and schooldays (*p* = 0.65). However, the participants woke up about 1.5 h later during holidays than on schooldays (t = 4.31, *p* < 0.01 and d = 1.24). Although TIB was significantly longer during holidays compared to schooldays (t = 4.26, *p* < 0.01, d = 1.23, and Δ = 72.30 min), there was no significant difference in TST, SE, and SOL (*p* = 0.40, *p* = 0.08, *p* = 0.59, respectively). Results of WASO are presented in Table 2. WASO was significantly higher on holidays than on schooldays (t = 2.41, *p* = 0.03, d = 0.69, and Δ = 39.72 min).

### 3.3. Sleep Parameters on Weekdays vs. Weekends

**Figure 1:**
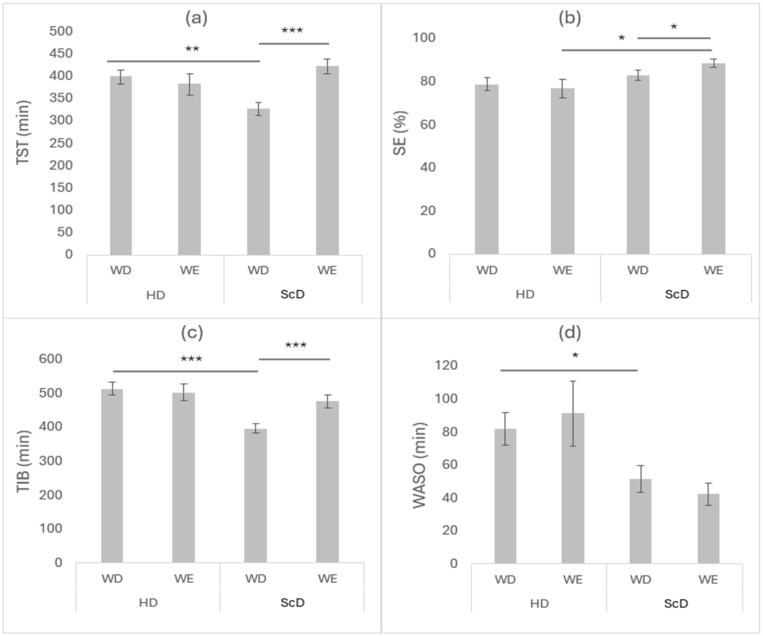
Mean ± standard error of (**a**) TST, (**b**) SE, (**c**) TIB, and (**d**) WASO on weekdays and weekends during holidays and schooldays. Abbreviations: HD = holidays, ScD = schooldays, SE = sleep efficiency, TST = total sleep time, WD = weekdays, WE = weekend. * *p* < 0.05 ** *p* < 0.01, and *** *p* < 0.001.

Two-way repeated-measures ANOVA tests revealed a significant effect of day (F_(1,11)_ = 10.3, *p* < 0.01, and ηp^2^ = 0.48) and a significant interaction (week × day) (F_(1,11)_ = 17.64, *p* < 0.01, and ηp^2^ = 0.62) on TST, but no significant effect of week was observed (F_(1,11)_ = 0.77, *p* = 0.40, and ηp^2^ = 0.07). On schooldays, the Bonferroni post hoc test showed that TST on weekdays was significantly shorter than that on weekends (*p* < 0.001, d = −1.64, and Δ = 95.42 min). On weekdays, participants slept less on schooldays than on holidays (*p* < 0.01, d = −1.01, and Δ = 72.46 min) (Figure 1).

Furthermore, repeated-measures ANOVA showed no significant main effect of day (F_(1,11)_ = 0.89, *p* = 0.37, ηp^2^ = 0.08) and week (F1,11) = 3.64, *p* = 0.08, ηp^2^ = 0.25), but identified a significant interaction for SE (F_(1,11)_ = 7.23, *p* = 0.02, ηp^2^ = 0.40). On schooldays, SE was higher on weekends compared to weekdays (*p* = 0.01, d = −0.88, and Δ = 5.71%), and it was also higher than SE on weekends during holidays (*p* = 0.03, d = 0.72, and Δ = 11.7%) (Figure 1).

The results of two-way repeated-measures ANOVA revealed a significant effect of week (F_(1,11)_ = 18.17, *p* < 0.01, ηp^2^ = 0.62) and day (F_(1,11)_ = 7.56, *p* = 0.02, ηp^2^ = 0.41) and a significant interaction (F_(1,11)_ = 10.27, *p* < 0.01, ηp^2^ = 0.48) for TIB. The Bonferroni test revealed that TIB on weekdays was significantly shorter than on weekends during schooldays (*p* < 0.001, d = −1.49, and Δ = 80.08 min). In the same vein, on weekdays, participants spent more time in bed on holiday than on schooldays (*p* < 0.001, d = −1.83, and Δ = 117.63 min) (Figure 1).

Moreover, statistical analysis revealed only a significant effect of week for WASO (F_(1,11)_ = 5.82, *p* = 0.03, ηp^2^ = 0.35). The Bonferroni post hoc test showed that on weekdays, WASO significantly increased during holidays compared to schooldays (*p* = 0.02, d = −0.77, and Δ = 30.48 min) (Figure 1). For SOL, no significant effect of day (F_(1,11)_ = 0.11, *p* = 0.75, ηp^2^ = 0.01), week (F_(1,11)_ = 0.89, *p* = 0.37, ηp^2^ = 0.08), or (day × week) interaction (F_(1,11)_ = 0.13, *p* = 0.72, ηp^2^ = 0.01) was observed.

### 3.4. Mid-Point of Sleep and Social Jetlag

The mid-point of sleep and social jetlag are presented in Table 3. The results showed no significant difference between holidays and schooldays for these variables, except for mean midsleep on weekdays, which was significantly later during holidays (t = 2.23, *p* < 0.05, and d = 0.65).

## 4. Discussion

To our knowledge, this is the first study to assess objectively (i.e., via actigraphy) weekday-to-weekend and schooldays-to-holidays sleep discrepancies in adolescent athletes, particularly basketball players in a naturalistic environment. The main findings were that during the two-week period (i.e., holidays and schooldays), our adolescent basketball players did not meet the minimum recommendations for sleep quality and quantity, with an average of 6 h 22 min of TST and about 82% of SE. Moreover, during schooldays, our athletes slept significantly less on weekdays compared to weekends and to weekdays on holidays. However, despite social jetlag being greater during schooldays compared to holidays, the difference was not statistically significant. In addition, during weekdays, athletes’ sleep was more fragmented (i.e., higher WASO) on holidays than schooldays.

Despite recommendations from the Centers for Disease Control and Prevention (CDC), the American Academy of Sleep Medicine (AASM), and the National Sleep Foundation (NSF) advocating for adolescents to obtain 8–10 h of sleep per night [13,63,67], our participants fell short of this recommendation, averaging only 6.37 h of nightly sleep duration. These findings align with previous studies indicating that the minimal sleep recommendation is often not met by adolescents [20]. Data of a meta-analysis of 41 surveys investigating sleep patterns subjectively in Australia, Europe, Asia, and the United States showed restricted sleep duration on an international scale and a tendency for higher rates of daytime sleepiness [20]. A similar pattern of inadequate sleep has also been documented in adolescent athletes [41,68,69,70,71]. In a recent study, nearly half of all adolescent athletes (196) included, both males (156) and females (40), and across all sport types (e.g., basketball, soccer, tennis), are sleeping less than 8 h per night [71]. Comparable sleep durations were noted among UK-based adolescent team-sport athletes (*n* = 2727, age ≈ 17 years), who averaged 7.7 h of sleep [68]. More alarmingly, studies based on objective sleep measures (i.e., sleep-EEG device and actigraphy) revealed that Swiss soccer players (*n* = 12, age ≈ 16 years) [69] and Asian athletes in shooting and track and field sprinting (*n* = 11, age ≈ 15 years) [70] experience even more severe sleep shortages, with averages of 7 and 5.5 h, respectively. In a recent narrative review, Mason et al. [37] reported that adolescent athletes achieve, on average, ≈6.3 h of sleep. This highlights a widespread trend of insufficient sleep among young athletes across different sports and regions. In the same way, during a 21-month follow-up study in 112 adolescent athletes, Milewski et al. [41] reported almost 80% of them were sleeping 7 h or less per night. It is noteworthy that in this study, authors suggested that less than 8 h of sleep per night was the strongest predictor of injuries. Indeed, over the supervised 21 months, 65% of this young athletes’ population who reported sleeping less than 8 h per night experienced at least one injury [41]. Recent studies have further illuminated the intriguing link between the incidence of musculoskeletal injuries and the sleep quantity and quality [39,72,73]. Importantly, regarding sleep quality, the results of our adolescent athletes showed that SE (81, 81%) did not reach the international recommendations of at least 85% [63]. Moreover, our findings indicated the presence of complaints of several episodes of awakenings during the night operationalized through a WASO (66.70 min) higher than the recommendations for good sleep quality (≤20 min) [63]. Our results are in line with previous studies reporting WASO higher than 20 min in adolescents athletes [72]. According to the literature, a WASO higher than 20 min indicates poor sleep quality and fragmented sleep, and may be associated with musculoskeletal injuries in athletes [63,72]. Thus, this is an important sleep variable to be taken into consideration in sports practice by the staff professionals, especially in adolescent athletes. Furthermore, we found in our study a significant association between greater eveningness preference (MEQ) and poorer sleep quality (PSQI). In this way, it has been reported that adolescents presenting the evening-type profile (E-types) showed a significantly higher rate of multiple night-wakings [55]. Moreover, previous studies reported that E-types were more prone to sleep complaints [74] and presented lower SEs [75], as measured subjectively (i.e., PSQI and the Epworth Sleepiness Scale) [74] and objectively (via actigraphy) [75], than M-types. Therefore, the poor sleep quality reported in our study could possibly be explained by the tendency toward eveningness preference and the evening chronotype profile of our adolescents. These data emphasize the necessity for the proactive management of sleep hygiene in adolescent athletes.

**Figure 2:**
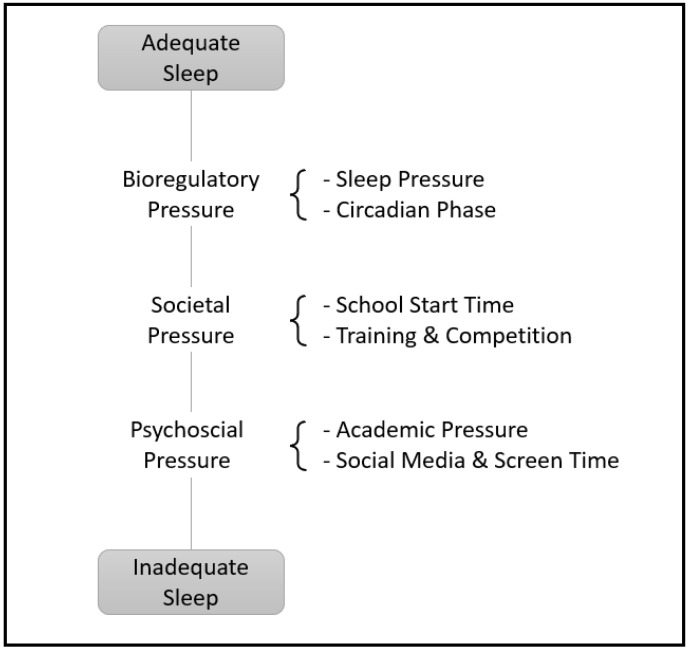
The perfect storm model adapted from previous studies [34,76,77].

Otherwise, our findings showed that when comparing schooldays and holidays (i.e., weekdays and weekend combined), our adolescent athletes spent more TIB (≈72 min) during holidays. This result is consistent with a previous study with the same sleep measurement duration (2 weeks) using subjective assessment tools [76]. The authors reported an increase in TIB by 88 min on holidays compared to the school period [76]. With BT not being significantly different between the two periods, this increase in TIB is mainly due to a later RT. In fact, previous studies also reported similar delays in RT during holidays compared to schooldays [76,77]. Interestingly, although TIB increased, no significant difference was reported between the school period and holidays in TST, SE, and SOL. This could be explained by the increase in WASO from the school period to holidays (39 min). Comparing these periods (with combining weekdays and weekend) revealed that even if adolescent athletes are spending more TIB, their sleep was fragmented, not letting them catch up on sleep debt; especially, during the school period, our adolescents had insufficient sleep (TST ≈ 6 h). A closer examination of the data to assess differences between weekdays and weekends over the two periods (HD and ScD) showed that TST on weekdays was significantly reduced compared to weekends during ScD (Δ = 95.42 min) and to weekdays during HD (Δ = 72.46 min), leading to a TST of 5 h and 26 min. These findings are consistent with numerous international [20] and national [42,78,79] surveys, highlighting the significantly reduced sleep duration among adolescents during schooldays. The changes observed in adolescents’ sleep patterns on weekdays during ScD might be attributed mainly to a restricted TIB on weekdays due to early school start times, which leads to early RT, shorter sleep durations, and a subsequent “sleep debt” that is carried over to weekends [76,80]. In this way, previous studies observed that students who attended delayed-start schools had an additional sleep time (>40 min) [80], they had fewer complaints about fatigue and daily sleepiness, and they had less difficulty concentrating and paying attention in classes, when compared to students who started classes early [76]. Moreover, at odds with our initial expectations, social jetlag was not significantly different between HD and ScD. This could be explained by the delayed BT reported in our results. In fact, adolescence is often marked by a shift toward later BT, a trend that has been consistently observed in surveys worldwide. These studies reported a progressively later BT on both school and non-school nights, as well as later RT on non-school or HD mornings as adolescents grow older [81,82]. Moreover, cross-sectional and longitudinal studies using objectively estimated sleep (via actigraphy) further confirm these findings [83,84]. These changes in adolescent sleep patterns are mainly caused, on one hand, by maturational development manifested by a circadian phase delay [85,86] and a greater robustness to increased sleep pressure (reduced adenosine accumulation) [87]. On the other hand, these changes are the results of societal and psychosocial factors such as social networks [88], increased “screen time” (i.e., television viewing, mobile phone, and internet use) [89] and bedtime autonomy, and growing academic workload [90]. In fact, it has been shown that adolescents delay bedtime to study, which comes at the expense of sleep [43]. Additionally, for an adolescent athletic population like ours, training and competition commitments are important contributing factors to the changes in their sleep patterns [37] (Figure 2).

## 5. Practical Applications

The management and the improvement of sleep in athletes is becoming an increasingly important area in sports science. According to a recent systematic review, there is a significant gap in both awareness and guidance concerning the essential role of sleep and the promotion of healthy sleep habits among adolescents [91]. Therefore, coaches and staff professionals need to be mindful of factors that disrupt sleep, such as overly packed schedules and early morning commitments [35]. Accordingly, one essential step to prevent sleep issues in athletes is to ensure that sports schedules, especially competitions and travel [92], are designed to allow adequate sleep opportunities. A recent consensus emphasizes the importance of integrating sleep screening and ongoing monitoring into adolescent athletes’ healthcare, particularly for those experiencing sleep difficulties [8]. While further research is needed to confirm the effectiveness of strategies like relaxation techniques and nutritional interventions, daytime napping appears to be a promising and cost-effective approach that has already yielded positive results on physical [9], cognitive [10], and sport-specific performance [11], as well as physiological response [93].

## 6. Limitations

First, a limited sample size and only one basketball team were used for this study considering that athletic populations are often difficult to access. Further, to reduce variance related to sex and age, the sample consisted of only male adolescents aged between 15 and 17 years old. Thus, these results cannot be systematically generalized to all populations, and larger studies are still needed to confirm the veracity of the present findings. Second, future research should consider using polysomnography (PSG), which is the gold standard for objective sleep measurements, providing more detailed information on sleep patterns, including sleep stages [94]. Indeed, it has been suggested that actigraphic measurements might overestimate WASO and therefore underestimate TST in adolescents [95]. Nevertheless, actigraphy remains a reliable, non-invasive, and cost-effective tool compared to PSG [94,96] and a practical means to obtain objective sleep data over 24 h and for durations of a few weeks (e.g., 2 weeks) in adolescents [77]. Especially, previous reports suggest that the prevalence of sleep deprivation may be more severe than what is indicated in subjective measures [95]. Third, physical activity and daily workload were not taken into consideration, even though these factors could affect adolescents’ sleep. Finally, future research could consider objective measures related to the circadian rhythm (e.g., melatonin, cortisol, DLMO) to gain more insights into the mechanistic aspects, particularly among the athlete population.

## 7. Conclusions

The finding of the present study showed that adolescent athletes are not meeting the minimum sleep recommendation in terms of quantity (i.e., TST) and quality (i.e., SE and WASO) on schooldays and holidays. Further, their sleep is even shorter on school weekdays compared to weekends and holiday weekdays. This study provides an understanding of adolescent athletes’ sleep hygiene and habits and underline the severity of inadequate sleep issues among this population. It is recommended that coaches, staff professionals, parents, and adolescent athletes be educated about the negative effects of suboptimal sleep on health, as well as on athletic and academic performances, and about strategies to promote sleep health and ameliorate adolescent athletes’ sleep patterns.

## Figures and Tables

**Table 1:** Sample characteristics (*n* = 12).

Age (Years)	Body Weight (kg)	Height (m)	BMI (kg/m^2^)	Body Fat (%)	Weekly Training Frequency	Years of Practice
15.58 ± 0.67	63.50 ± 8.42	1.72 ± 0.09	21.34 ± 1.98	14.67 ± 3.63	4.17 ± 0.39	7.33 ± 1.30

Notes: Values represented in mean ± SD. Abbreviations: BMI = body mass index.

**Table 2:** Mean ± SD of sleep parameters.

	Holidays	Schooldays	*p*	Cohen’s d
Bedtime (h:min)	00:50 ± 00:42	00:41 ± 00:39	0.65	0.14
Rise time (h:min)	09:13 ± 01:11	07:42 ± 00:45	<0.01	1.24
Time in bed (min)	508.46 ± 67.63	436.17 ± 49.57	<0.01	1.23
Total sleep time (min)	390.50 ± 65.06	374.46 ± 46.73	0.40	0.25
Sleep efficiency %	77.88 ± 12.46	85.73 ± 7.13	0.08	−0.55
Sleep onset latency (min)	25.44 ± 38.28	13.92 ± 19.12	0.59	0.27
Wake after sleep onset (min)	86.56 ± 51.33	46.84 ± 24.26	0.03	0.69

**Table 3:** Midsleep during weekdays and weekend, and social jetlag on holidays and schooldays.

	Holidays	Schooldays	*p*	Cohen’s d
Midsleep on weekdays (h:min)	4:54 ± 1:05	3:58 ± 0:51	<0.05	0.65
Midsleep on weekend (h:min)	5:17 ± 1:01	4:38 ± 1:37	0.32	0.30
Social jetlag (h:min)	0:22	0:40	0.69	0.10

Notes: Values represented in mean ± SD.

## Data Availability

The data sets examined in this study can be obtained from the corresponding author upon reasonable request.
